# PARP Inhibitors in Small-Cell Lung Cancer: Rational Combinations to Improve Responses

**DOI:** 10.3390/cancers13040727

**Published:** 2021-02-10

**Authors:** Erik H. Knelson, Shetal A. Patel, Jacob M. Sands

**Affiliations:** 1 Dana-Farber Cancer Institute, Boston, MA 02215, USA; erik_knelson@dfci.harvard.edu; 2 Department of Medicine, University of North Carolina School of Medicine, Chapel Hill, NC 27599, USA; shetal_patel@med.unc.edu

**Keywords:** SCLC, PARP, DDR, ICB, synthetic lethality, SLFN11, STING

## Abstract

**Simple Summary:**

Small-cell lung cancer carries a dismal prognosis with few long-term treatment options. The enzyme poly-(ADP)-ribose polymerase (PARP), which functions to repair DNA breaks, has emerged as a promising therapeutic target, with modest response rates in early clinical trials prompting investigation of predictive biomarkers and therapeutic combinations. This review summarizes the development and testing of PARP inhibitors in small-cell lung cancer with an emphasis on developing treatment combinations. These combinations can be divided into three categories: (1) contributing to DNA damage; (2) inhibiting the DNA damage response; and (3) activating the immune system. An evolving classification of small-cell lung cancer subtypes and gene expression patterns will guide PARP inhibitor biomarker identification to improve treatments for this challenging cancer.

**Abstract:**

Despite recent advances in first-line treatment for small-cell lung cancer (SCLC), durable responses remain rare. The DNA repair enzyme poly-(ADP)-ribose polymerase (PARP) was identified as a therapeutic target in SCLC using unbiased preclinical screens and confirmed in human and mouse models. Early trials of PARP inhibitors, either alone or in combination with chemotherapy, showed promising but limited responses, suggesting that selecting patient subsets and treatment combinations will prove critical to further clinical development. Expression of SLFN11 and other components of the DNA damage response (DDR) pathway appears to select for improved responses. Combining PARP inhibitors with agents that damage DNA and inhibit DDR appears particularly effective in preclinical and early trial data, as well as strategies that enhance antitumor immunity downstream of DNA damage. A robust understanding of the mechanisms of DDR in SCLC, which exhibits intrinsic replication stress, will improve selection of agents and predictive biomarkers. The most effective combinations will target multiple nodes in the DNA damage/DDR/immune activation cascade to minimize toxicity from synthetic lethality.

## 1. Introduction

Small-cell lung cancer (SCLC) is a high-grade neuroendocrine malignancy with a poor prognosis that accounts for 13% of all lung cancer diagnoses [1,2]. First-line treatment for extensive-stage SCLC (ES-SCLC) is often effective, with a response rate of more than 60% to platinum-based chemotherapy, but prior to recent first-line advances, median overall survival was less than 11 months [2,3]. Immune checkpoint blockade (ICB) using inhibitors of the programmed cell death protein and its ligand (PD-1/PD-L1) initially showed promise in the third-line setting, and inclusion into first-line platinum-based therapy has demonstrated an overall survival benefit, becoming the new standard of care [4,5,6,7] with particular improvement in durable responses. Until recently, topotecan has been the only option approved by the United States Food and Drug Administration (FDA) in the second-line setting but has not been widely utilized due to concerns about toxicity and only modest efficacy [8,9,10]. Despite this, multiple randomized studies with a topotecan control arm have been negative, highlighting the resistant disease state [11,12,13] after prior platinum-based therapy. One of the negative studies that failed to meet its primary overall survival endpoint was a recent combination of lurbinectedin and doxorubicin compared to a control arm of either topotecan or CAV (cyclophosphamide, doxorubicin, vincristine) [14], which followed prior accelerated FDA approval of single-agent lurbinectedin based upon impressive data in small-cell lung cancer from a basket trial [15]. National Comprehensive Cancer Network guidelines include multiple regimens that may be considered in the second-line setting and beyond, but clinical trial is one of the three preferred regimens, highlighting the need for more effective treatments [16].

SCLC is a transcriptionally active disease with common (up to 90%) loss-of-function genomic alterations in the tumor suppressor genes *TP53* and *RB1*, creating further genomic instability by preventing arrest of the cell cycle for important DNA repair [17,18,19,20]. This suggests the potential for synergy with treatments that disrupt replication enough to halt the process and lead to apoptosis. One such approach, poly-(ADP)-ribose polymerase (PARP) inhibitors, have been a compelling class of drugs in the ongoing efforts to improve outcomes in this cancer that has been so resistant to other treatment options. Overexpression of PARP1 in SCLC further suggests therapeutic potential for PARP inhibitors [21].

Recurrent, targetable genomic alterations have not been identified in SCLC, but epigenetic and gene expression studies have led to the description of four distinct molecular subtypes defined by transcriptional regulators [22]. Subtyping of SCLC may offer an opportunity for better identification of treatment options with a higher likelihood of generating durable responses and will likely be an important component of prospective studies, including those evaluating PARP inhibitors and combinations.

PARP inhibitors represent a therapeutic class that has become an important treatment option for multiple tumor types. Although there is evidence of response, PARP inhibitors are not currently part of the treatment armamentarium for SCLC, and single-agent efficacy is limited. There is substantial ongoing investigation incorporating PARP inhibitors into the treatment of SCLC, and the following sections outline the mechanisms and rationale for these promising therapeutic combinations.

## 2. PARP Inhibitor Mechanism of Action

**Figure 1:**
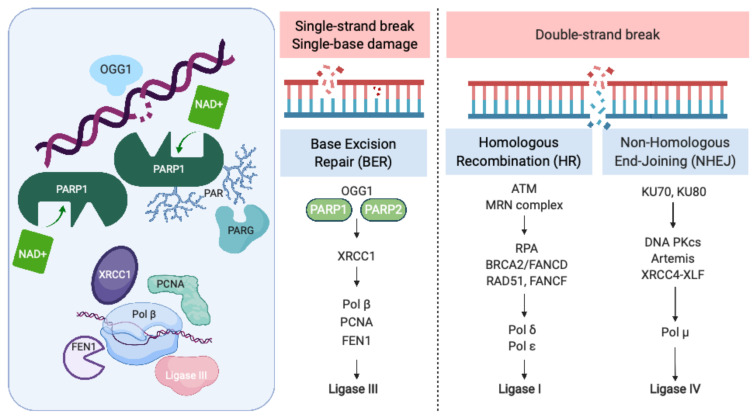
The Role of PARP in the DNA Damage Response. PARP = poly-(ADP)-ribose polymerase, OGG1 = 8-oxoguanine glycosylase, XRCC1 = X-ray repair cross-complementing protein 1, Pol β = DNA polymerase beta, PCNA = proliferating cell nuclear antigen, FEN1 = flap endonuclease 1, ATM = ataxia telangiectasia, mutated, MRN complex = Mre11 + RAD50 + NBS1/nibrin, RPA = replication protein A, BRCA2 = FANCD1 breast cancer susceptibility gene and DNA repair enzyme, Pol δ = DNA polymerase delta, Pol ε = DNA polymerase sigma, KU70/80 = lupus Ku autoantigen protein p70/p80, DNA PKcs = DNA-dependent protein kinase, catalytic subunit, XRCC4 = X-ray repair cross-complementing protein 4, XLF = XRCC4-like factor, and Pol μ = DNA polymerase mu. Created with BioRender.com; accessed on 21 January 2021.

Recognition and repair of DNA damage form an essential cellular function mediated by a number of interconnected pathways termed the DNA damage response (DDR; Figure 1). PARP enzymes are a family of proteins that function in recognition and repair of DNA breaks, chromatin remodeling, and transcriptional regulation [23]. PARP 1 and 2 enzymatic function is activated by binding single-strand DNA breaks (SSB) and involves poly-ADP ribosylation (PARylation) of various substrates and recruitment of proteins that mediate DNA repair (Figure 1). PAR groups are subsequently metabolized by Poly-(ADP)-ribose glycohydrolase (PARG) and other enzymes as part of coordinated dePARylation critical to effective DNA repair [23]. In the absence of SSB repair by PARP1, the replication fork stalls and double strand breaks occur prompting repair via homologous recombination (HR) or non-homologous end joining (NHEJ). If DSBs are not correctly repaired, replication aberrancies such as mutations, deletions, chromosomal translocations, and amplifications can occur resulting in cell death, senescence or malignant transformation. PARP inhibitors were initially developed to sensitize tumor cells to standard treatments such as chemotherapy or radiation, which induce DNA damage [24]. However, the observation that tumor cells with defects in HR are highly sensitive to single-agent PARP inhibition accelerated their clinical development [25,26]. The activity of PARP inhibitors in patients with *BRCA1* or *BRCA2* mutant cancers was the first clinical demonstration of synthetic lethality for cancer therapy [27]. In this setting, by inhibiting PARP catalytic activity and trapping PARP on DNA, PARP inhibitors stall replication machinery leading to DNA double strand breaks (DSB). In the absence of BRCA1 or BRCA2, these breaks cannot be repaired by HR (Figure 1). Several PARP inhibitors are currently approved or in clinical trials. In addition to differences in their selectivity for PARP 1/2, these agents differ in their PARP trapping function, with talazoparib being the most potent [28]. Further studies in tumors without HR deficiency suggest that PARP inhibitors could have a broader role in cancer therapy [29].

PARP was initially identified as a potential therapeutic target in SCLC through seminal work by Byers et al., who performed unbiased proteomic analysis of cell lines using reverse-phase protein arrays (RPPA) to identify proteins that were differentially expressed in SCLC compared with non-small-cell lung carcinoma (NSCLC) [21]. PARP1 transcript and protein levels were significantly elevated in SCLC cell lines compared to NSCLC. Increased PARP1 protein expression was also confirmed by immunohistochemical (IHC) analysis of tissue microarrays. Notably, several other components of the DDR pathway were increased in SCLC, including the checkpoint kinases CHK1 and CHK2, the ataxia telangiectasia related protein ATR, and the DNA-dependent protein kinase catalytic subunit DNA PK_cs_, which may be important to maintain cell viability in light of high replication stress (Figure 1). Treatment of a series of lung cancer cell lines with AZD2281 (olaparib) demonstrated that SCLC lines were significantly more sensitive to PARP inhibition than other histologic subtypes of lung cancer. Combining PARP inhibition with chemotherapy further decreased tumor cell viability.

These observations led to the initial studies of PARP inhibitors in SCLC as single agents (Table 1). In a phase I trial of talazoparib, 23 patients with SCLC were treated at the recommended phase II dose of 1.0 mg daily [30]. Two patients had a partial response, for an objective response rate (ORR) of 9% with a duration of response of 12.0 and 15.3 weeks. Both patients with an objective response had a platinum-free interval of 6 months or less. An additional four patients had stable disease, for a clinical benefit rate of 26% at 16 weeks. The UK STOMP trial examined the role of olaparib in the maintenance setting, but failed to show an improvement in progression-free survival (PFS) [31].

## 3. Biomarkers of Response to PARP Inhibitors in SCLC

Since only a subset of SCLC patients appears sensitive to PARP inhibition, identification of predictive biomarkers has been an important focus of translational research. Clinical trials have attempted to identify correlative markers of response. Based on their preclinical work Owonikoko et al. examined DNA-PKcs expression as a biomarker but did not observe a correlation with veliparib activity in their phase II trial [32]. Although elevated serum lactate dehydrogenase (LDH) levels and male gender are poor prognostic markers, in the veliparib arm, these correlated with improvement in PFS in multivariable analysis. Mutations in DNA damage response genes such as *BRCA1*, *BRCA2*, *ATM*, or *ATR* (Figure 1) are not frequently seen in SCLC. However, homologous recombination deficiency (HRD) assays have been used to identify *BRCA1*/*2* wildtype ovarian cancer patients with sensitivity to PARP inhibition. Using three different measures of HRD, Lok et al. analyzed a series of SCLC cell lines to determine if HRD predicted response to PARP inhibition [33]. While they did not observe any correlation between HRD scores and response to PARP inhibitors, gene expression analysis demonstrated that high levels of *schlafen family member 11* (*SLFN11*) transcript did correlate with PARP inhibitor sensitivity. SLFN11 has been identified as critical for SCLC cell line and patient-derived xenograft (PDX) response to chemotherapy [34,35], as well as a potential biomarker for PARP inhibitor response using unbiased screens in SCLC cell lines and PDXs [33,35]. SLFN11 is a protein that is recruited to sites of DNA damage, inhibits HR, and activates a replication-stress response. High levels of SLFN11 have been correlated with enhanced response to PARP inhibitors in many [33,34,35,36,37] but not all [38,39] SCLC trials and preclinical models. Furthermore, using clustered regularly interspaced short palindromic repeats (CRISPR) based gene editing, deletion of *SLFN11* was found to confer resistance to talazoparib [33]. Importantly for clinical translation, a SLFN11 IHC H-score predicted sensitivity of SCLC PDXs to PARP inhibition [34]. A bimodal expression pattern of *SLFN11* transcript levels was observed in SCLC from The Cancer Genome Atlas (TCGA) dataset [33].

Using the NCI-60 database to identify genomic correlates of sensitivity to talazoparib across multiple tumor types, Murai et al. also identified *SLFN11* among the top-ranking genes [37]. They observed that deletion of *SLFN11* conferred resistance to PARP inhibition but found that ATR inhibition could overcome this resistance. An integrated proteomic and transcriptomic analysis of SCLC PDX models also identified SLFN11 protein levels as predictive of response to PARP inhibition [40]. Additionally, low ATM and high E-cadherin expression correlated with sensitivity to PARP inhibition. Treatment with cisplatin or PARP inhibitors reduced SLFN11 expression in cell line models, raising the question of dynamic changes in this marker in response to prior therapy. Using gene expression derived from PDX models, Farago et al. identified an inflammatory gene signature *(CEACAM1*, TNFSF10, OAS1, TGIF1) that selected for sensitivity to olaparib + temozolamide. Markers of epithelial-to-mesenchymal transition (EMT) and high *MYC* target gene expression correlated with resistance. Collectively, these studies demonstrate that high SLFN11 expression is a promising biomarker for sensitivity to PARP inhibitor activity in SCLC, but prospective validation is needed and integration of multiple markers may improve predictive ability. SLFN11 expression is being studied prospectively as a biomarker in a randomized phase II clinical trial of talazoparib as maintenance therapy with atezolizumab in patients with ES-SCLC (SWOG1929, NCT04334941). Recent preclinical work argues that SCLC subtype can also influence response to PARP inhibitors, with expression of the transcription factor *POU2F3* sensitizing to PARP inhibitors [41].

## 4. PARP Inhibitors Combined with Chemotherapy

**Figure 2:**
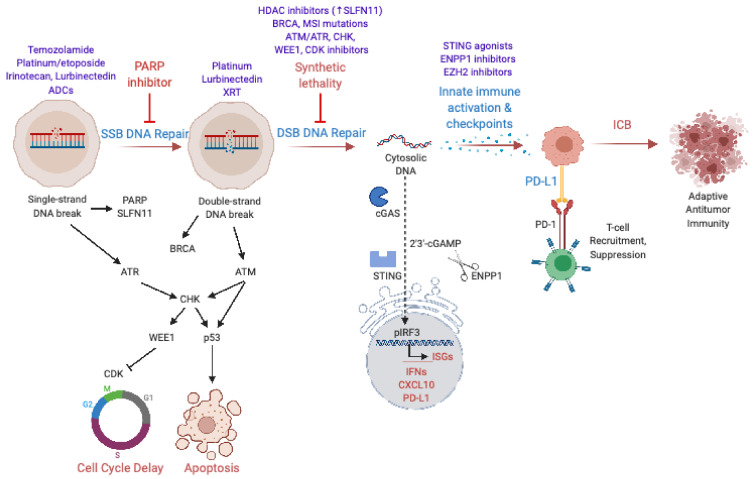
PARP Inhibitor Combinations: Enhancing Response in SCLC. PARP = poly-(ADP)-ribose polymerase, ADCs = antibody–drug conjugates, SSB = single-strand DNA break, DSB = double-strand DNA break, CDK = cyclin-dependent kinase, XRT = radiation therapy, HDAC = histone deacetylase, MSI = microsatellite instability, ssDNA = single-strand DNA, ISGs = Interferon-stimulated genes, and ICB = immune checkpoint blockade. Created with BioRender.com; accessed on 29 January 2021.

Given limited single-agent activity, a number of preclinical and clinical studies have examined combinations of PARP inhibitors with chemotherapy, radiation, and targeted therapies to enhance therapeutic benefit (Figure 2, Table 1). Several groups have demonstrated that PARP inhibition can potentiate the activity of platinum-based chemotherapy in SCLC cell lines and xenografts [21,42,43]. Owonikoko et al. tested the combination of veliparib with cisplatin (75 mg/m^2^) and etoposide (100 mg/m^2^ on days 1–3) in a phase I/II randomized clinical trial (ECOG-ACRIN 2511) in patients with ES-SCLC [32,44]. The recommended phase II dose for veliparib in combination with cisplatin and etoposide was determined to be 100 mg twice daily on days 1–7. Patients treated with veliparib had a median PFS of 6.1 months (95% CI, 5.9 to 6.7) relative to 5.5 months for placebo (95% CI, 5.0 to 6.1). Overall survival was 8.9 months (95% CI, 8.3 to 11.3) in patients receiving placebo relative to 10.3 months (95% CI 8.9 to 12.0) with the addition of veliparib (stratified HR, 0.83; 80% CI 0.64 to 1.07; *p* = 0.17). Patients were stratified by sex and serum LDH levels. Male patients with high LDH levels derived benefit in PFS (HR 0.34; 80% CI 0.22 to 0.51), but no difference in OS by strata was observed. The combination was tolerable, with higher rates of lymphopenia and grade 3 or 4 neutropenia seen with the addition of veliparib. The combination of veliparib with carboplatin (AUC = 5) and etoposide (100 mg/m^2^ on days 1–3) has also been studied [45]. The recommended phase II dose for veliparib in this study was 240 mg twice daily for days 1–14, due to excess hematologic toxicity seen with continuous dosing. A randomized phase II study was performed with three arms: (A) carboplatin/etoposide + veliparib followed by veliparib, (B) carboplatin/etoposide + veliparib followed by placebo, and (C) carboplatin/etoposide + placebo followed by placebo. Median PFS in arm A was 5.8 months (80% CI 5.6 to 6.8), arm B 5.7 months (5.6 to 5.8) and 5.6 months (5.1 to 6.7) for arm C. Similarly, no significant differences in OS were observed.

PARP inhibitor and chemotherapy combinations have also been examined in patients with relapsed disease after platinum-based chemotherapy (Table 1). Temozolomide (TMZ) is an oral alkylating agent, previously demonstrated to have single-agent activity in SCLC [46]. TMZ methylates the O^6^ position of guanine, ultimately leading to DSBs. O^6^ methylguanine-DNA methyltransferase (MGMT) is involved in repair of these lesions; therefore, silencing of *MGMT* expression by promoter methylation has been correlated with improved clinical response to TMZ. Given that PARP proteins also have a role in repair of these lesions, it was hypothesized that the combination of PARP inhibitors and TMZ could have synergistic activity. Using talazoparib, Lok et al. evaluated the activity of TMZ and PARP inhibition in several SCLC models, demonstrating synergistic tumor growth inhibition, particularly in high *SLNF11*-expressing models [33]. *MGMT* expression did not correlate with sensitivity to TMZ + talazoparib. Murai et al. similarly observed synergistic activity for talazoparib and TMZ in SCLC models with high *SLFN11* expression [37]. Two phase II studies in relapsed SCLC patients have evaluated the combination of TMZ and PARP inhibition. Pietanza et al. performed a randomized, double-blind, placebo-controlled study of veliparib (40 mg twice daily, days 1 to 7) or placebo and TMZ (150–200 mg/m^2^/day, days 1 to 5) on a 28-day cycle [36]. The primary endpoint of the study was 4-month PFS, with no significant differences observed between TMZ/veliparib (36%) and TMZ/placebo (27%, *p* = 0.19). Median PFS was 3.8 and 2.0 months (log-rank *p* = 0.39, HR 0.84; 95% CI 0.56 to 1.25) for the TMZ/veliparib and TMZ/placebo arms, respectively. OS was also similar between the 2 arms. ORR was higher for the combination of TMZ/veliparib (39%) versus TMZ/placebo (14%), in both platinum-sensitive and platinum-refractory patients. Biomarker analysis was performed for PARP-1 and SLFN11 expression by IHC. No association with PARP-1 expression and clinical outcomes was observed. SLFN11-positive tumors (H-score cutoff ≥ 1) treated with TMZ/veliparib had improved PFS (5.7 vs. 3.6 months, *p* = 0.009) and OS (12.2 vs. 7.5 months; *p* = 0.014). Notably, the authors highlighted that a low dose of veliparib was used in this study and veliparib has lower PARP trapping activity, both of which could have contributed to limited efficacy.

Using the more potent PARP inhibitor olaparib in combination with TMZ, Farago et al. performed a phase I/II study in relapsed SCLC [39]. To facilitate biomarker analysis and mechanistic studies, a co-clinical trial with PDXs was performed. At the recommended phase II dose of olaparib (200 mg twice daily, day 1–7) and TMZ (75 mg/m^2^, day 1–7 of 21 days cycle), the ORR was 41%, with a median duration of response of 5.3 months. Across all dose levels, PFS was 4.2 months (95% CI, 2.8 to 5.7) with a median OS of 8.5 months (95% CI, 5.1 to 11.3). A phase II study of continuous talazoparib with intermittent low-dose TMZ (NCT03672773) in relapsed/refractory SCLC is ongoing. Additional studies are evaluating the combination of PARP inhibitors with agents that induce DNA damage such as pegylated SN-38, the active metabolite of irinotecan, an inhibitor of topoisomerase I activity (NCT04209595, Table 2).

Antibody–drug conjugates (ADCs) are another class of therapeutic that acts by inducing DNA damage selectively in tumor cells after targeting to tumor-specific antigens. The first ADC to enter clinical development for SCLC targeted the Notch inhibitory protein delta-like ligand 3 (DLL3), identified as enriched on SCLC cells with impaired Notch signaling [47]. The DLL3 ADC, Rovalpituzumab tesirine (Rova-T), showed promise in preclinical work and early trials [48], but ultimately failed to meet primary endpoints in phase II/III trials [12,49]. A phase I trial of an ADC targeting the tumor-associated calcium signal transducer Trop2 showed an acceptable safety profile and promising results with an ORR of 14% and clinical benefit in 34% of heavily pretreated patients with SCLC [50]. Further studies are ongoing, and ADCs may be of particular benefit in combination with other treatments including PARP inhibitors.

In addition to chemotherapy combinations, PARP inhibitors can also sensitize SCLC models to ionizing radiation [42,51]. Laird et al. noted that talazoparib is a more potent radiosensitizer than veliparib, suggesting that PARP trapping ability may play a role in sensitization to radiation. Talazoparib treatment led to increased DSBs compared to veliparib. Interestingly, radiosensitization was observed irrespective of SLFN11 expression. Several early phase trials are examining the combination of PARP inhibition and radiation in ES-SCLC (NCT03532880, NCT04170946, Table 2). Potential toxicity to normal tissues with these combinations is a concern and will be carefully evaluated in these studies.

## 5. Synthetic Lethality Downstream of PARP Inhibitors

In addition to synergy with chemotherapy and radiation, several preclinical studies have suggested that combinations with DDR inhibitors could enhance the therapeutic potential of PARP inhibitors in SCLC. These approaches aim to target multiple nodes of the DDR response to prevent resistance and promote synergistic antitumor activity. Since PARP inhibition prevents repair of single-strand DNA breaks, which subsequently progress to DSBs at stalled replication forks, PARP inhibitors are most effective when DSB repair is impaired. This strategy, referred to as synthetic lethality, was originally developed in the setting of *BRCA* germline mutations in ovarian cancer patients and holds particular appeal in SCLC, where defining mutations in *RB1* and *P53* combine with elevated tumor mutational burden from tobacco exposure to generate additional replication stress and dependence on DNA repair mechanisms [52,53]. SCLC is not associated with germline *BRCA* mutations, and global microsatellite instability in SCLC is rare. However, a “DNA-repair score” was shown to correlate with response to PARP inhibition in preclinical work using SCLC PDXs [54]. This prognostic score includes canonical DNA repair genes such as *PARP*, *BRCA*, *ATM*, *ATR*, *CHK*, *RAD50*, *53BP1*, *MSH2*, and *FANC* (Figure 1) [54], several of which can also be inhibited pharmacologically (Figure 2). Early phase clinical trials have opened for SCLC patients targeting the DNA damage response (Table 2), including for AZD1775 targeting WEE1 (NCT02593019, NCT02688907), VX-970 targeting ATR (NCT02157792, NCT02487095), and Prexasertib targeting CHK (NCT02735980) [52]. While these specific trials do not include PARP inhibitors, combination studies with PARP inhibition are also being developed.

CHK1 is a protein kinase that plays an important role in DNA damage-dependent cell cycle arrest, particularly in TP53-deficient tumors. CHK1 protein expression is increased in SCLC patient tumors [21,55]. Combination therapy with the CHK1 inhibitor LY2606368 and cisplatin or olaparib enhanced tumor regression and survival in mouse SCLC models [55].

WEE1 is a kinase involved in S phase and G_2_-M progression, by phosphorylating CDK1/2 and allowing for DNA repair prior to mitotic entry. Targeting WEE1 with inhibitors such as AZD1775 compromises DNA damage checkpoints, particularly in cancer cells that may be more dependent on the G_2_-M checkpoint. Lallo et al. studied the combination of olaparib and AZD1775 in SCLC PDXs and observed activity in both chemotherapy sensitive and resistant models [56]. This combination is being evaluated in a trial for patients with refractory solid tumors, including SCLC (NCT02511795).

Preclinical work using cell lines and patient specimens suggests that treatment with histone deacetylase (HDAC) and enhancer of zeste homology 2 (EZH2) inhibitors can restore epigenetically suppressed SLFN11 expression [57], suggesting potential synergy with PARP inhibition. In the majority of SCLC with low SLFN11 expression, resistance to PARP inhibition may be overcome by pharmacologic ATR inhibition [37], further supporting the role for DDR synthetic lethality in enhancing response to PARP inhibitors in SCLC.

## 6. Combining PARP and Immune Checkpoint Inhibition

ICB has been incorporated into the first-line treatment of SCLC [6,7]. Combining ICB and PARP inhibitors may offer synergy because of molecular signaling pathways linking cytosolic DNA to PD-L1 expression. DNA-sensing pathways, which evolved to protect against bacteria and viruses, also recognize self-DNA released from the nucleus when DDR is suppressed [58]. Double-stranded DNA is recognized by the enzyme cyclic GMP-AMP synthase (cGAS), which produces the cyclic dinucleotide second messenger 2’3’-cGAMP, activating the Stimulator of Interferon Genes (STING) pathway, which upregulates interferon stimulated genes, including PD-L1. PARP inhibitors have been shown to activate STING and upregulate PD-L1 across cancer models regardless of *BRCA* mutation status, leading to synergy with PD-L1 inhibitors in preclinical mouse studies [59,60,61,62,63]. In SCLC, preclinical data suggest that synergy between PARP inhibitors and PD-L1 checkpoint inhibition may depend on intact tumor cell STING and innate immune activity downstream of cytosolic DNA released after PARP inhibition [64]. A phase II trial in relapsed SCLC combining durvalumab 1500 mg every 4 weeks with olaparib 300 mg twice a day showed an ORR of 10.5% (two patients out of nineteen) [65]. The treatment combination was well tolerated, with expected cytopenias from PARP inhibition but no evidence of overlapping toxicity. Of note, both responders exhibited an inflamed phenotype with CD8+ T cells contacting tumor cells in a pretreatment biopsy [65]. Co-mutation status and/or histology may influence response to combined PARP and immune checkpoint inhibition in SCLC, as one of the responders had a *BRCA* mutation that may have sensitized to PARP inhibition, and the other had *EGFR*-mutant transformed SCLC. Post-treatment biopsies confirmed increases in PD-L1 expression after PARP inhibition in 6/9 paired cases. However, these increases failed to correlate with T-cell infiltration. The disappointing response rates in this trial are similar to a previous phase II basket study including patients with relapsed SCLC that used the same doses of olaparib and durvalumab but with a 4-week olaparib run-in period [66]. These early results suggest that additional mechanisms suppress antitumor immunity in SCLC. The phase II trial of rucaparib + nivolumab in platinum-sensitive SCLC (NCT03958045) may identify a clinical context with residual disease where these agents are more effective [67].

Additional immune checkpoints, such as CTLA-4 (cytotoxic T-lymphocyte-associated protein 4), which binds to CD80/CD86, may suppress antitumor immunity downstream of PARP inhibition. A number of trials combining PARP and CTLA-4 inhibitors are currently underway, including a phase I trial of thoracic radiation combined with durvalumab +/− tremelimumab or olaparib in ES-SCLC after first-line chemotherapy (NCT03923270) [67]. Antibody dependent cellular cytotoxicity (ADCC) represents another promising approach to unleash antitumor immunity. Preclinical studies identified BMS-986012 as an antibody that can bind the tumor cell-specific ganglioside FucGM1, leading to ADCC [68]. This compound is currently being tested in phase I/II trials (NCT02247349, NCT02815592), either as part of first-line treatment for ES-SCLC alongside chemotherapy or in the relapsed setting alongside nivolumab. As the designs of these trials suggest, targeting multiple steps in DNA damage response concurrently (see Figure 2) may ultimately prove successful.

## 7. Restoring Tumor Cell Inflammatory Signaling to Enhance PARP Inhibitor Response

The majority of SCLC are “immune deserts” with minimal infiltration by CD8+ effector T cells [22]. However, a subset of non-neuroendocrine tumors demonstrates enhanced inflammatory infiltrates and markers of innate immunity including restored STING expression [69,70]. As suggested by the phase II data for durvalumab + olaparib [65], and confirmed in elegant preclinical work [41], the non-neuroendocrine inflamed subtype may represent a biomarker for response to this combination. To expand the patient population that can benefit from the combination of DDR inhibition and ICB, novel approaches to restore tumor cell inflammatory pathways are sorely needed. The neuroendocrine stress response inhibits inflammation, so strategies that target neuroendocrine lineage commitment could elicit antitumor immunity. Reversing EZH2 epigenetic programing to de-repress antigen presentation and tumor cell STING expression represents one promising approach [70,71]. Indeed, EZH2 levels are higher in SCLC than any other tumor type in TCGA [72], and EZH2 inhibitors can restore *SLFN11* expression to potentially improve response to PARP inhibitors [34]. The combination of EZH2 and PARP inhibitors was effective in preclinical models of ovarian cancer [73], and is being developed in SCLC, where both approaches have shown promise as monotherapies [21,74]. Restoring tumor suppressive *NOTCH1* or inhibiting the Notch suppressive protein DLL3 to alter neuroendocrine differentiation could have similar effects, as recent evidence suggests that phenotype switching can uncover therapeutic vulnerabilities [41]. Targeting negative regulators of DNA-sensing including ectonucleotide pyrophosphatase/phosphodiesterase family member 1 (ENPP1), the enzyme that cleaves the STING second messenger 2′3′-cGAMP [75], may also potentiate the effects of DDR inhibition. ENPP1 can also metabolize PAR downstream of PARP in the DNA damage response [76]. While inhibiting PARylation and dePARylation simultaneously may seem counterproductive, both processes cooperate in DNA damage repair, and their concurrent inhibition shows promise in preclinical cancer models [77]. Combinations that disrupt coordinated DNA damage repair are more likely to stimulate innate antitumor immunity and response to immune checkpoint blockade.

## 8. Orthogonal Approaches

The past decade has seen many advances in SCLC management, culminating in the adoption of ICB into first-line treatment [6]. Previously, second-line treatment was limited to topoisomerase inhibitors, but this has recently expanded to include lurbinectedin and a host of promising clinical trials [78]. Many of these trials include PARP inhibitors, either alone or in combination as outlined in prior sections. Investigational targets outside of DNA damage, repair, and antitumor immunity include receptor tyrosine kinases (RTKs) and their ligands, which can be inhibited with monoclonal antibodies or tyrosine kinase inhibitors (TKIs). Disrupting tumor angiogenesis by targeting the vascular endothelial growth factor (VEGF) has proven effective in other cancers but failed in clinical trials for SCLC [79]. Preclinical work suggests combining VEGF monoclonal antibodies with checkpoint blockade in SCLC [80], and there is also interest in inhibiting VEGF alongside PARP [28]. A phase II trial evaluating olaparib in combination with the VEGF TKI cediranib has enrolled patients with SCLC (NCT02498613). This combination previously proved successful in extending PFS from PARP inhibition in recurrent platinum-sensitive ovarian cancer [81]. Preclinical studies have also identified fibroblast growth factors (FGF) and their receptors as therapeutic targets in SCLC, where approximately 6% of patients harbor amplifications in FGFR1 [53]. Signaling pathways downstream of RTKs offer additional targets. Preclinical data demonstrate an increase in PI3K/mTOR activity following PARP inhibition in SCLC models, providing rationale for combination therapy with PARP inhibitors plus PI3K/mTOR inhibitors [82]. A phase I/II trial is currently underway evaluating the mTOR inhibitor vistusertib in combination with the Bcl-2 inhibitor navitoclax in relapsed SCLC (NCT03366103). In theory, combined inhibition of growth factor signaling and PARP could enhance clinical response [28,78].

## 9. Conclusions

PARP inhibitors are a compelling class of drugs in the treatment of SCLC, with a mechanism of action that takes advantage of genomic instability and loss-of-function *TP53*/*RB* genomic alterations that challenge the cells’ ability to repair DNA. Single-agent trials have demonstrated only modest results that do not yet warrant a role in the treatment armamentarium. Combination therapy such as temozolomide + olaparib has improved outcomes, and many other combinations are in progress or development. Biomarkers to identify patient subsets likely to respond to PARP inhibitors and/or combinations with synergistic mechanisms of action are required in the further development of PARP inhibitors as effective treatments for SCLC. SLFN11 and other components of the DDR pathway, perhaps combined in an expression signature, represent putative predictive biomarkers for PARP inhibitors, though prospective validation will be required.

SCLC subtyping provides a framework for future drug development. As new therapeutic options are prospectively evaluated within the context of identified subtypes of SCLC, an increasing opportunity exists to further define predictive biomarkers. For example, *POU2F3* expression may be as valuable in identifying tumors susceptible to PARP inhibition as *SLFN11* expression [41,83]. The emerging “inflamed” subtype may also demonstrate improved responses to PARP inhibitors in combination with ICB [41,65,84], since preclinical data suggest that downstream DNA-sensing pathways remain intact in some tumors [70] and could amplify the effects of impaired DDR. Epigenetic strategies to reverse subtype-specific gene expression patterns may also uncover vulnerability to PARP inhibitors. HDAC inhibitors to increase *SLFN11* expression [57] and EZH2 inhibitors to reverse neuroendocrine immunosuppression [71] are two notable examples.

Multiple compounds are in development to synergize with PARP inhibitors. In this review, we organized PARP combinations by mechanism of synergy (Figure 2): DNA damage, repair of DNA breaks/synthetic lethality, and immune activation. We predict that the most successful combinations will include compounds from multiple categories, analogous to vertical pathway inhibition downstream of RTKs. However, unlike combinations of TKIs, PARP inhibitor combinations may prove more tolerable for patients since toxicities are less likely to overlap and synergy will be most pronounced in SCLC cells with impaired DDR, allowing for dose decreases to minimize side effects. Though SCLC prognosis remains grave, clinical and translational advances in recent years offer hope of combining PARP inhibitors with agents that impair DDR and activate antitumor immunity to improve response rates and survival. Enthusiasm for PARP inhibitor combinations raises hopes that synthetic lethality and restored antitumor immunity, therapeutic strategies with great success in other cancers, can benefit patients with SCLC.

## Figures and Tables

**Table 1:** Studies including PARP inhibitors in SCLC with outcomes data.

Study Population	Drug(s)	Response Rate	PFS (Months)	OS (Months)	Unique Trial Data
Patients with ≤1 prior treatment regimen^30^	Talazoparib	9%	11.1 weeks		
First-line ES-SCLC^32^	CE + veliparib vs. CE + placebo	71.9% vs. 65.6%	6.1 vs. 5.5	10.3 vs. 8.9	Elevated LDH and male gender correlated with benefit
First-line ES-SCLC^45^	(A) CE+ veliparib -> veliparib (B) CE + veliparib -> placebo (C) CE + placebo -> placebo	77% 59.3% 63.9%	5.8 5.7 5.6	10.1 10.0 12.4	
Relapsed ES-SCLC^36^	TMZ + veliparib vs. TMZ + placebo	39% vs. 14%	3.8 vs. 2.0	8.2 vs. 7.0	SLFN11 positive tumors prolonged PFS and OS
Relapsed ES-SCLC^39^	TMZ + olaparib	41.7%	4.2	8.5	Co-clinical PDX trial
Relapsed ES-SCLC^65^	Durvalumab + olaparib	10.5%	1.8	4.1	Inflamed phenotype→ response
Relapsed ES-SCLC^66^	Durvalumab + olaparib	5.3%			Olaparib run in

PFS = progression-free survival, OS = overall survival, ES-SCLC = extensive-stage small-cell lung cancer, CE = cisplatin/etoposide, LDH = lactate dehydrogenase, TMZ = temozolamide, SLFN11 = schlafen family member 11, and PDX = patient-derived xenograft.

**Table 2:** Ongoing studies in SCLC.

Study Population	Drug(s)	Phase	Unique Trial Data	Trial Number
ES-SCLC	Talazoparib + Atezolizumab maintenance	II	Prospective study of SLFN11 expression	NCT04334941
Relapsed/refractory ES-SCLC	Intermittent low-dose TMZ + continuous Talazoparib	II	Previous trials used intermittent talazoparib	NCT03672773
SCLC	PLX038 (Pegylated SN-38) + rucaparib	I/II	Potential enhancement in DNA damage from formulation of irinotecan metabolite	NCT04209595
ES-SCLC	Olaparib + low-dose radiotherapy	I	Maintenance therapy for stable disease after first-line chemotherapy	NCT03532880
ES-SCLC	Talazoparib + consolidative thoracic XRT	I	Maintenance therapy for stable disease after first-line chemotherapy	NCT04170946
Relapsed/refractory ES-SCLC	AZD1775 (WEE1)	II	Single-arm study	NCT02593019
Relapsed/refractory ES-SCLC	AZD1775 (WEE1)	II	Single-arm study; CDKN2A or MYC mutation required	NCT02688907
ES-SCLC	VX-970 (ATR) + CE or cisplatin (platinum resistant)	I	Flexible enrollment with first-line chemotherapy or relapsed/refractory disease	NCT02157792
Relapsed/refractory ES-SCLC	VX-970 (ATR) + topotecan	I/II		NCT02487095
Relapsed/refractory ES-SCLC	Prexasertib (CHK)	II		NCT02735980
Relapsed/refractory ES-SCLC	AZD1775 (WEE1) + olaparib	1b		NCT02511795
ES-SCLC	Rucaparib + nivolumab	II	Maintenance therapy for stable disease after first-line chemotherapy	NCT03958045
ES-SCLC	Thoracic radiation combined with durvalumab +/− (tremelimumab + olaparib)	I	Maintenance therapy for stable disease after first-line chemotherapy	NCT03923270
Relapsed/refractory ES-SCLC	BMS-986012 +/− nivolumab	I/II		NCT02247349
ES-SCLC	BMS-986012 + CE	I/II	First-line therapy	NCT02815592
Relapsed/refractory ES-SCLC	Olaparib + cediranib (VEGF)	II	Correlation with DNA repair gene expression	NCT02498613
Relapsed/refractory ES-SCLC	Vistusertib (mTOR) + Navitoclax (Bcl-2)	I/II	On treatment biopsy	NCT03366103

ES-SCLC = extensive-stage small-cell lung cancer, SLFN11 = schlafen family member 11, TMZ = temozolamide, and CE = cisplatin/etoposide.
